# Effect of Different Molecular Weight Hyaluronic Acids on Skim Milk Functional Properties

**DOI:** 10.3390/foods13050690

**Published:** 2024-02-24

**Authors:** Rutvi Joshi, Suresh G. Sutariya, Prafulla Salunke

**Affiliations:** Dairy and Food Science Department, South Dakota State University, Brookings, SD 57007, USA; rutvijitendrakumar.joshi@jacks.sdstate.edu (R.J.); suresh.sutariya@jacks.sdstate.edu (S.G.S.)

**Keywords:** hyaluronic acid, rheology, functional, microstructure

## Abstract

Hyaluronic acid (HA), a naturally occurring polysaccharide with recognized health benefits, has gained approval for use in the food industry as a food additive, ingredient, and health supplement in numerous countries. HA can increase viscosity in solutions and is available commercially in various molecular weights (MW) depending on end applications. Nevertheless, no research has explored the impact of different MW HAs on functionality, rheological properties, and texture-building benefits in the dairy product matrix wherein they are incorporated. Therefore, the objective of this study was to evaluate how varying MWs of HA—specifically 8 kDa, 320 kDa, 980 kDa, and 2550 kDa at 0.25% (*w*/*w*) concentration—impact rheological characteristics, functional attributes, heat stability, protein stability, protein structure, and protein fractions within skim milk. The addition of HA led to an increase in the apparent viscosity of all samples. A higher G″ value over G′ values for all HA samples was observed in frequency sweep, indicating the absence of interparticle interactions between HA particles. Protein stability and heat stability were significantly lower for 980 kDa and 2550 kDa HA as compared to the control and 8 kDa HA samples. As the MW increased, WHC, emulsion properties, and foaming stability notably increased. However, reversed results were found in the case of foaming activity. Moreover, no significant changes were observed in the percent area of individual protein fractions and the hydrodynamic diameter of protein particles. This study would help to understand the effect of HA when incorporated in dairy products for water binding or enhancement in viscosity-based applications.

## 1. Introduction

Hyaluronic acid (HA), also known as Hyaluronate and Hyaluronan, is a naturally existing mucopolysaccharide made up of repeating units of β4-glucuronic acid and β3-N-acetylglucosamine [1]. Nowadays, HA is mostly produced through animal tissue extraction, mainly from rooster combs or through the microbial fermentation of *Streptococcus* bacteria. Several disadvantages are associated with the product’s quality, safety, and cost when producing HA using the above-mentioned approaches. First, the polymer obtained from animal tissues may have variable quality and low quantitative yield and can carry allergenic animal protein along with polymer as an additional compound. Second, the animal protein from the polymer can be eliminated using extra processing steps of purification that increase the total production time, increasing the product cost. Third, since *Streptococcus* species are known for their pathogenicity, the production approach through bacterial fermentation poses the risk of product contamination with secreted exotoxins by bacteria and prevents its use in food applications. The addressed challenges were tackled by the genetic engineering of bacterial strains of *Bacillus subtilis*, *Lactobacillus lactis*, or *Corynebacterium glutamicum*, and this resulted in the production of a food-grade HA polymer with Generally Recognized As Safe (GRAS) status as per FDA 2020 [2,3,4,5,6].

The most interesting property of HA polymer is its hydrophilicity. HA molecules are negatively charged due to the presence of carboxyl groups of glucuronic acid in the structure. Therefore, HA molecules have the ability to bind with large amounts of water molecules through intra-molecular hydrogen bonds and can form a highly viscous gel. However, the increase in the hydrodynamic volume of HA polymer depends mainly on its concentration and the composition of the matrix [5,7]. Because of its moisture-binding property, its applications have been widely explored in biomedical, pharmaceutical, and cosmetic applications [5].

The commercial application of HA in bakery products as an egg white substitute was found long ago in 1942, even before its first use in medical and cosmetic products in the years 1960 and 1979, respectively. In 2011 and 2014, the Japanese Health Food and Nutrition Food Association and the Korean Ministry of Food and Drug Safety approved HA to be used as a food additive, respectively [8]. Afterwards, countries including China and the European Union accepted the usage of HA as a food ingredient. In the USA, Canada, Italy, and Belgium, HA is promoted to be used as a dietary supplement [9,10].

The global acceptance of HA and its water binding ability represents a huge market potential for dairy and food products in the near future as the application of polysaccharides in milk and food products that contain milk proteins is increasing with the purpose of modifying the viscosity and textural attributes of these products. However, limited research studies are available that have explored the functional benefits of HA in dairy and food applications. There is a huge potential for the oral delivery of HA through foods and as nanocarriers to deliver other health-benefiting bioactive compounds [11]. Wang et al. [12] formulated and characterized casein and HA emulsion gels for use in food-based 3D printing material. Sutariya and Salunke [9] studied the impact of HA (1550 kDa) at different concentrations on milk rheological, acid and rennet gelation, and protein stability, and the results showed an obvious increase in viscosity as the concentration of HA increases, with a negative impact on heat stability, protein phase separation, and gelation properties.

Moreover, HA’s impact on rheological properties and functional properties such as water holding capacity, foaming capacity, foaming stability, emulsion activity, and emulsion property in milk when used along with kappa-carrageenan at different concentrations of 0.1 and 0.25% and ratios of 85:15, 70:30, and 50:50 for each concentration were also analyzed [13]. Zhong et al. [14] reported an interaction between whey protein isolate and HA as an effect of pH and mixing ratio. Chon et al. [15] explored the properties of kefir with HA addition and found that the addition of HA did not affect the physicochemical and organoleptic properties of kefir and that HA could be used as a health-improving agent, especially in skin-damage and muscle. HA has been used in other foods, for example, Zajac et al. [6] reported improved rheology and water-binding properties in smoked homogenized sausages after adding HA. Tan et al. [11] recently reviewed the use of HA as a nano-delivery vehicle for food bioactive compounds and reported that these carriers have been developed with an improved solubility, stability, and bioavailability of polyphenols, lipids, and vitamins. 

The functional attributes of products containing HA would depend upon the MW or chain length of the HA used [5]. In biomedical applications, HA with MW higher than 10 kDa was found to be appropriate to use in products used in ophthalmology, orthopedics, cosmetics, and tissue engineering [16,17,18], whereas low MW HA (≤5 kDa) was reported to be useful in formulating product substances that aid angiogenesis, prevent tumor progression, and actuate pro-inflammatory mediator expression [5,19,20]. Most of the previous studies concentrated on using HA with a single MW. Commercially up to 3000 kDa HA is available. HA with a molar mass of as high as 10,000 kDa can be produced [18]. Since HA is available in a range of MWs, its distinctive functional characteristics associated with viscoelastic and rheological properties would play a significant role in dairy and food applications. However, there are no studies available wherein the effect of HA with different MW within the milk environment has been investigated. It would be important to understand the effect of different MW HA in milk since milk-based products demand different applications including viscosity enhancement, gelation, emulsification, foaming etc. 

Therefore, the objective of this study is to understand the interaction of different MW HA with various physicochemical and functional properties of skim milk: (i) rheological behavior in the milk environment, (ii) protein stability during storage, (iii) heat stability during processing at ultra-high temperature, (iv) water holding capacity, (v) emulsifying activity and emulsion stability under processing conditions, (vi) foaming capacity and stability during storage, and (vii) modifications in protein structure, size, and individual fractions. This study will help the dairy industry to understand the effect of HA with different MW on functional and rheological characteristics of skim milk, paving a way for it to be used in dairy products wherein water retention/binding is desired such as yogurt, beverages, and processed cheese products.

## 2. Materials and Methods

### 2.1. Materials 

Three different lots of skim milk was purchased from a local grocery store (Great Value, Walmart, Brookings, SD, USA). The sodium azide was obtained from Fisher Scientific (Fair Lawn, NJ, USA). The food-grade HA (Sodium hyaluronate) powder samples having three different molecular weights, 10 kDa, 200 kDa, 800 kDa, were purchased from the Stanford Chemicals Company (Lake Forest, CA, USA). The research grade HA powder with a high molecular weight of >2500 kDa was obtained from the HA works (Bedminster, NJ, USA). According to the certificate of analysis provided by suppliers, the actual MW of HA powder samples were 8 kDa, 320 kDa, 980 kDa, and 2550 kDa. The appearance of the different HA powders was white with a fine crystal texture, and the purity was >95%. 

### 2.2. Methods

#### 2.2.1. Experimental Design and Statistical Analysis

The composition of each pasteurized skim milk was analyzed. The skim milk from each lot was divided into five parts, in which HA of 8 kDa, 320 kDa, 980 kDa, and 2550 kDa were added at 0.25% concentration *w*/*w*. Skim milk without HA was considered as a control for the study. The prepared samples were further analyzed for viscosity, frequency sweep, protein stability, heat stability, water holding capacity, emulsion activity, emulsion stability, foaming capacity, foaming stability, particle size, calcium concentration, protein fractions, and microimaging of the protein structure. The experiments were performed in three replicates for all samples using a different lot of skim milk in each replicate, and each analysis was carried out in triplicate. Statistical analysis between the samples was examined in the R software version 4.2.3 (R Foundation for Statistical Computing, Vienna, Austria) using one-way ANOVA with a 95% confidence level, and mean comparison was performed using Tukey’s test where the differences were considered significant when *p*-values were <0.05.

#### 2.2.2. Skim Milk Analysis

The pasteurized skim milk samples’ compositional analysis (fat, lactose, and total solids) was achieved using the DairySpec FT (Bentley Instruments Inc., Chaska, MN, USA). The total protein content was analyzed using the standard Kjeldahl method described by Hooi et al. [21]. The pH was determined using a pH meter (Hanna edge blu, Smithfield, RI, USA). 

#### 2.2.3. Skim Milk Sample Preparation with Hyaluronic Acid 

HA powders with a MW of 8 kDa, 320 kDa, 980 kDa, and 2550 kDa were added at 0.25% *w*/*w* concentration into the skim milk at ~5 °C and mixed using a lab-scale high shear homogenizer (Polytron^®^pt 2500 E, Kinematica AG, Malters, Switzerland) at 25,000 rpm for 3 min. After mixing, it was ensured that HA dissolved completely without forming any visible lumps. If any lumps were observed in the prepared samples, they were discarded, and samples were prepared again. Skim milk without the addition of HA was a control. Sodium azide at a concentration of 0.02% *v*/*v* was added and mixed to each sample as a preservative. The samples were stored in a refrigerator (~5 °C) and allowed to hydrate for 12 h. After hydration, samples were analyzed for the viscosity profile and frequency sweep. Subsequently, any changes in the pH of samples were analyzed and adjusted to a pH of 6.7 either using the 1 M HCl or 1 M NaCl by the drop-wise addition method where the milk was continuously stirred at 25 °C. These samples were further analyzed for protein stability, heat stability, and functional properties.

#### 2.2.4. Rheological Behavior of Skim Milk

The viscosities of the control and HA-treated samples were determined using a method described by Sutariya et al. [22] with slight modifications. Viscosity was determined at 10 °C using a rotating rheometer (MCR 92, Anton Paar GmbH, Graz, Austria) using bob and cup assembly. Samples were mixed gently, and 60 mL was poured into a cup (C-CC39, 42.010 mm) fitted with a rheometer and a concentric cylinder attachment (CC39, 38.690 mm), operated by the Anton Paar Rheo Compass 1.20 system. The viscosity was measured over the shear rate profile from 10 to 1000 s^−1^ where every 3 s the shear rate was increased by 10 s^−1^. The gap between the bob and cup was kept 5 mm. The viscous behavior of samples was evaluated using the following equation of the Power Law model:

(1) $$\sigma =K*\gamma^{n}$$

where σ is the shear stress (Pa), K is the consistency coefficient (Pa s^n^), γ is the shear rate (s^−1^), and n is the dimensionless flow behavior index that defines the shear thinning or shear thickening properties of a fluid. 

To understand the time-dependent behavior of HA polymers in the non-destructive deformation environment, a frequency sweep test was performed using the rheometer over an angular frequency range of 11.5–150 rad/s, at a constant shear strain of 0.5%, at a temperature of 10 °C throughout the experiment.

#### 2.2.5. Protein Stability by Gravimetric Phase Separation

The protein stability of samples during storage was determined using the method described by Sutariya and Salunke [9] with slight modifications. The 50 mL sample was transferred into a 50 mL volumetric cylinder and stored undisturbed in the refrigerator at ~5 °C for 48 h to allow the gravimetric phase separation of proteins as an impact of HA polymers. The top of the volumetric cylinder was covered with plastic wrap. The visual phase separation of the samples was constantly checked for the initial 7 h when stored. After 48 h, the top 15 mL sample between the middle-separated phase layer and top layer were collected and analyzed for total protein content by Hooi et al. [21], and % protein phase separation was calculated using the following equation.

(2) $$\%Protein\;phasese\;paration=\frac{\left(Protein\;in\;top\;15\;mL\;layer-Protein\;in\;control\;sample\right)}{Protein\;in\;control\;sample}\;*100$$

#### 2.2.6. Heat Stability by Heat Coagulation Time Test (HCT)

The heat stability of samples was determined as per the method described by Kasinos et al. [23] and Sutariya and Patel [24] with some modifications. First, a 5 g sample was transferred into the screw-capped glass vials. Then, the tubes were placed in a rocker and immersed in an oil bath (high-temperature oil bath, Narang Scientific works Pvt Ltd., New Delhi, India) containing mineral oil at a temperature of 140 ± 3 °C with constant agitation. The heat coagulation time was noted as the time elapsed between the samples immersed in the mineral oil bath and an inception of visible clots.

#### 2.2.7. Water Holding Capacity (WHC)

The water holding capacity was determined as per the method described by Li et al. [25] with slight modifications. First, the prepared test samples were placed in a hot water bath maintained at 30 °C for 15 min. Subsequently, 10 mL of the sample was transferred to a 10 mL graduated centrifuge tube, and the tubes were centrifugated using a Sorvall ST plus series centrifuge (ThermoFisher Scientific, Karlsruhe, Germany) at force of 2000× *g* at 30 °C for 15 min. After centrifugation, the amount of water bounded with milk matrices in sediment was recorded (in mL). Water holding capacity was calculated as the percentage ratio of sediment volume (in mL) to the initial sample volume (in mL). 

#### 2.2.8. Oil Emulsifying Activity and Emulsion Stability

The oil emulsifying activity and emulsion stability were evaluated as per the method described by Benvenutti et al. [26] with slight adaptations. The samples and sunflower oil (Hy-Vee, Brookings, SD, USA) were first kept in a water bath maintained at 30 °C for 15 min, and 70 parts of the test samples were mixed with 30 parts of the sunflower oil, and oil-in-water emulsion was obtained by mixing the samples using a lab-scale high sheer homogenizer (Polytron^®^pt 2500 E, Kinematica AG, Malters, Switzerland) at a speed of 15,000 rpm for 2 min at 30 °C. Subsequently, 10 mL was transferred into the 10 mL volumetric graduated centrifuge tube and centrifugated at 30 °C for 15 min at force of 2000× *g* using the Sorvall ST plus series centrifuge (ThermoFisher Scientific, Karlsruhe, Germany). Emulsion volume was recorded, and oil emulsion activity was calculated as the percentage ratio of emulsion volume (in mL) to the initial sample volume (in mL). 

After the oil emulsion activity test was completed, the same tubes samples were pasteurized in a hot water bath maintained at 80 °C for 30 min, followed by cooling at 30 °C for 15 min. The tubes were recentrifuged at 30 °C for 15 min at force of 2000× *g* using the Sorvall ST plus series centrifuge (ThermoFisher Scientific, Karlsruhe, Germany), and emulsion volume was recorded. Emulsion stability was calculated as the percentage ratio of emulsion volume (in mL) to the initial sample volume (in mL).

#### 2.2.9. Foaming Capacity and Foaming Stability 

The foaming properties were analyzed using the method described by Cano-Medina et al. [27] with minor modifications. The test samples were warmed in a water bath at 30 °C for 15 min. Subsequently, 100 mL test samples were filled into the 250 mL graduated plastic beaker. The solutions were agitated in the same container using a high shear homogenizer (Polytron^®^pt 2500 E, Kinematica AG, Malters, Switzerland) at speed of 15,000 rpm for 3 min. The sample volume immediately after 1 min of agitation was noted. The foaming capacity was reported as:

(3) $$Foaming\;capacity\;\%=\frac{\left(Sample\;volume\;after\;1\;min\;of\;foaming-initial\;sample\;volume\right)\;mL}{\left(Initial\;sample\;volume\right)\;mL}\times 100$$

To study the foaming stability, the prepared samples for foaming capacity were stored further in a water bath maintained at 30 °C, and any reduction in the foam volume during the interval of 0.5, 1, 1.5, 2, 4, 6, 8, 16, 24 h of storage period was noted. The foaming stability was expressed as:

(4) $$Foaming\;stability\;\%=\frac{\left(Sample\;volume\;after\;x\;min\;of\;foaming-initial\;sample\;volume\right)\;mL}{\left(Initial\;sample\;volume\right)\;mL}\times 100$$

where, *x* = 0.5, 1, 1.5, 2, 4, 6, 8, 16, 24 h.

#### 2.2.10. Particle Size Analysis

An average hydrodynamic diameter of samples was determined using a Litesizer^TM^ 500 particle size analyzer (Anton Paar GmbH, Graz, Austria). Samples were diluted with glass distilled water in a ratio of 1:100 by following the serial dilution method. 1 mL of the diluted sample was transferred to a disposable cuvette and further analyzed for average particle size at 25 °C, in automatic measurement angle mode. An average particle size was expressed as hydrodynamic diameter (µm) in software.

#### 2.2.11. Capillary Gel Electrophoresis (CGE)

The individual milk protein fractions were determined using CGE as per the method described by Salunke et al. [28]. First, the samples were diluted to 1% protein using HPLC grade water, and 10 µL of diluted samples were mixed with 85 µL of sample buffer (Beckman Coulter, Fullerton, CA, USA) and 5 µL of β-mercaptoethanol (Fisher Scientific, Fair Lawn, NJ, USA) in a microvial. All samples were mixed thoroughly and heated at 90 °C for 10 min followed by cooling to room temperature prior to CGE run. CGE was performed using a Beckman P/ACE MDQ capillary electrophoresis machine (Beckman Coulter, Fullerton, CA, USA) mounted with a UV detector set at 214 nm. In accordance with the approach used by Salunke et al. [28], the separation was carried out using a 50-mm bare fused silica capillary at a constant voltage of 15 kV at 25 °C temperature and 20 bar pressure. The major five casein fraction (αS1-CN, αS2-CN, β-CN, K-CN, γ-CN) and two serum protein fraction (α-LA and β-LG) peaks were identified and calculated as a percentage of the total area by comparing the migration time of treatment samples with the control (skim milk) as well as by comparing them to the results presented by Salunke et al. [28].

#### 2.2.12. Determination of Calcium Concentration Using the EDTA Complexometric Titration Method

The soluble calcium concentration in the skim milk and HA added skim milk samples was determined [29,30,31]. 10 mL aliquots of the samples, both skim milk and HA-added hyaluronic acid, were transferred into 10.4 mL, 16 × 76 mm polycarbonate capped centrifuged bottles (Beckman Coulter, Inc., Brea, CA, USA, cat. 355603). Subsequently, the samples were centrifugated at 21,500× *g* at 20 °C in an ultracentrifuge (Optima MAX-E, Beckman Coulter, Inc., Brea, CA, USA) equipped with a fixed angle rotor (MLA-55, Beckman Coulter, Inc., Brea, CA, USA) for a duration of 90 min. This resulted in the separation of milk samples into supernatant (serum) and precipitation layers. The serum samples were then carefully transferred and stored in plastic tubes before proceeding with titration. For titration, one milliliter of serum samples was diluted by adding 50 mL of glass-distilled water. Subsequently, 3 mL of 8 M NaOH was added into the diluted sample to elevate the pH to ≥12.5. The samples were kept aside for 5 min with periodic swirling to facilitate the precipitation of any magnesium present in the solution. After that, 2–3 drops of a 0.5% *w/v* Patton-Reeder indicator (calconcarboxylic acid) (Sigma Aldrich, St. Louis, MO, USA) solution, prepared in 1 M NaOH, were added into the sample prior to titration. The samples were titrated using a 0.01 M EDTA until color change occurred, transitioning from pink to blue. The amount of EDTA required for titration was recorded. Here, moles of EDTA utilized during the titration is equivalent to the moles of calcium originally present in the serum sample. Hence, the mass of soluble calcium (mg) present in serum samples was determined using the following equation:

(5) $$Calcium\;concentration\left(\frac{mmol}{Liter}\right)=\left[Molarity\;of\;EDTA\;\times \;volume\;EDTA\;used\left(mL\right)\;÷\;sample\;volume\left(mL\right)\right]*1000$$

The quantification of total calcium concentration in skim milk was performed following the identical procedure as previously described. The mass of insoluble calcium (present in the precipitate) was calculated by subtracting the concentration of serum calcium from the total calcium concentration.

#### 2.2.13. Protein Microstructure Observation Using Confocal Laser Scanning Microscopy (CLSM)

For microscopic examination, proteins were labeled with fluorescein 5(6)-isothiocyanate (FITC) (Sigma Aldrich, St. Louis, MO, USA). 5 mL of skim milk and HA-added skim milk samples were mixed with 75 μL of 0.2% *w/v* FITC solution prepared in acetone. The resulting mixtures were vortexed for 5 s to ensure thorough mixing. Subsequently, two drops of these prepared samples were deposited onto microscopic slides and placed in a refrigerator at ~4 °C for a minimum duration of 1 h. This cooling period allowed for the evaporation of acetone and the binding of dye with the protein in sample. After that, the sample area was overlaid with a cover slip, and microscopic observations were conducted using an Olympus FV 1200 Fluoview confocal Scanning Laser Microscope (Olympus, Tokyo, Japan) using an air-cooled Ar/Kr laser with excitation wavelengths set at 488 nm and utilized a 10× objective lens. Representative images are reported. 

## 3. Results and Discussions

### 3.1. Composition of Skim Milk

The results of the compositional analysis of pasteurized skim milk are presented in Table 1. The mean pH value of skim milk was 6.65, which did not differ significantly upon adding different MW of HA, which means that the addition of HA does not impact acidity in milk [6,9].

### 3.2. Rheological Behavior of Skim Milk

**Figure 1:**
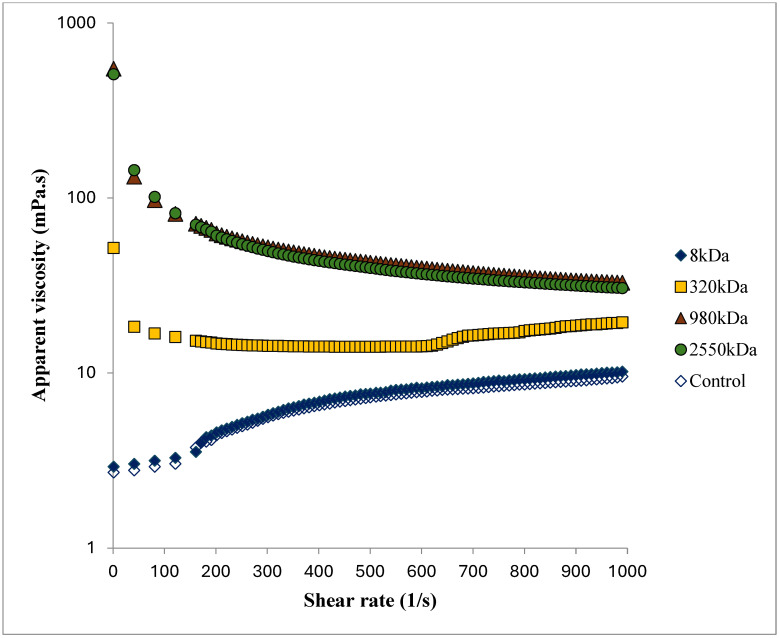
Viscosity profile of skim milk samples treated with different molecular weight of HA at 0.25% *w*/*w* concentration. Error bars for the standard error of mean (n = 3) not displayed for the better visual clarity of the graph.

The addition of different molecular weight HA into skim milk produced a notable change in the skim milk samples’ viscosity profile, as illustrated in Figure 1. The results indicated that the apparent viscosity increased as the molecular weight of HA increased, with the exception of the 8 kDA treatment sample. An increased apparent viscosity was observed for the 320 kDa, 980 kDa, and 2550 kDa treatment samples.

Analysis of the viscosity profiles (behavior index n and the consistency coefficient K (Pa s^n^)) using the power law model was carried out, and the results are displayed in Table 2. There was no significant (*p* < 0.05) change found in the K value for the control, 8 kDa, and 320 kDa treatment samples. K values for the higher molecular weight (980 kDa, and 2500 Kda) were statistically similar (*p* > 0.05), though they had higher and significantly different values than the other samples (control, 8 kDa, and 320 kDa). As molecular weight increased, flow behavior index n values were found to be decreasing, which indicates non-Newtonian behavior or shear thinning behavior in the sample. No significant difference was observed between the n values of the control and 8 Kda treatment samples. The n value decreased significantly for 320 kDa treatment as compared to the control and 8 kDa treatment. The lowest n values were observed for increasing MW HA sample (980 kDa and 2550 kDa) with no significant difference (*p* > 0.05).

**Figure 2:**
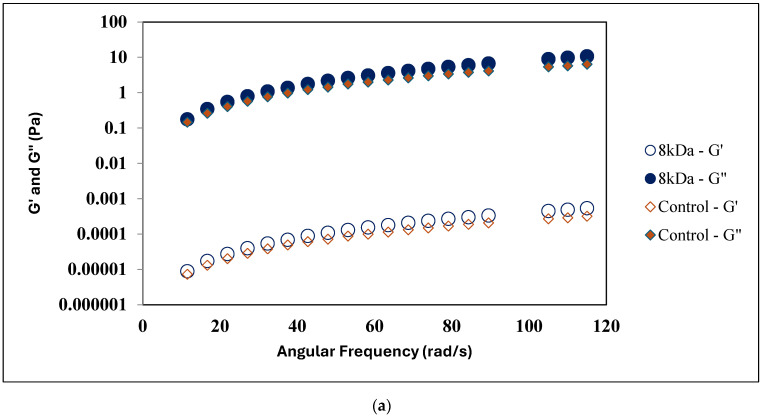

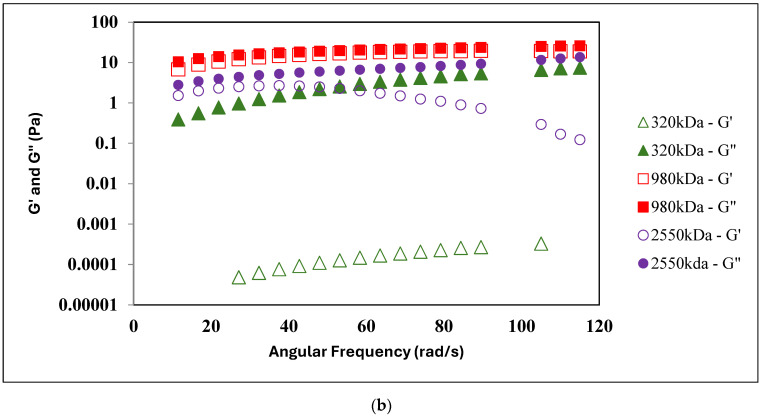
Frequency sweep test. Storage modulus (G′) and loss modulus (G″) as a function of skim milk samples treated with different molecular weight HA at 0.25% *w*/*w* concentration, (**a**) 8 kDa HA, Control (**b**) 320 kDa HA, 980 kDa HA, 2550 kDa HA. Error bars for the standard error of mean (n = 3) not displayed for the better visual clarity of the graph.

A frequency sweep test was carried out to understand the interaction of different MW HA. Figure 2a,b illustrates the frequency sweep results of the control and treatment samples (8 kDa, 320 kDa, 980 kDa, and 2550 kDa HA). Frequency sweep results for the control, 8 kDa HA, and 320 kDa HA samples show higher G″ values over G′ values, which indicates their viscoelastic liquid behavior without forming a gel network. For the 980 kDa treatment sample, G″ and G′ values were very close and remained linear to each other for the complete frequency range, indicating the onset of the formation of inter-polymer interactions. A similar trend in G″ and G′ values were obtained in a whole milk sample treated with 0.25% concentration of 1550 kDa HA [9]. However, an increase in MW to 2550 kDa HA showed lower storage modulus (G′) and loss modulus (G″) as compared to the 980 kDa HA treatment sample—though G″ > G′, both values were found to be nearer at a lower frequency range (<47.9 rad/s), and G′ declined at a higher frequency range (>47.9 rad/s), showing that as the oscillation rate increased, the structural rearrangement and interaction of polymer became ineffectual and formed a temporary three-dimensional network in the liquid milk system [32]. None of the samples represented a higher storage modulus (G′) over loss modulus (G″), which indicates the absence of HA’s interconnected polymer interaction and an inability to form a strong viscoelastic-solid gel network in the milk environment when HA was added at 0.25%. However, as per Sutariya and Salunke [9], when 1505 kDa HA’s concentration increased to 0.5% *w*/*w*, it formed a stable viscoelastic solid gel network. The formation of a gel-network is highly dependent on polymer concentration and MW, where the higher viscosity and shear thinning behavior of a higher MW HA sample (980 kDa HA & 2550 kDa HA) is attributed to increased water retention primarily via a certain level of intrapolymer interaction in the solution [9,32]. Additionally, it is important to note that the frequency sweep test was performed in triplicate for each sample. The G′ and G″ values obtained during the second and third runs were compared to those from the first run. Results from each replication revealed that pre-mixing the 8 kDa and 320 kDa samples prior to analysis had no impact on the viscoelastic liquid structure as the values remained consistent across all replicates. In contrast, the 980 kDa and 2550 kDa HA treatment samples yielded inconsistent results, suggesting large structural deformations due to a weak viscoelastic gel network within the milk environment.

### 3.3. Protein Phase Separation 

**Figure 3:**
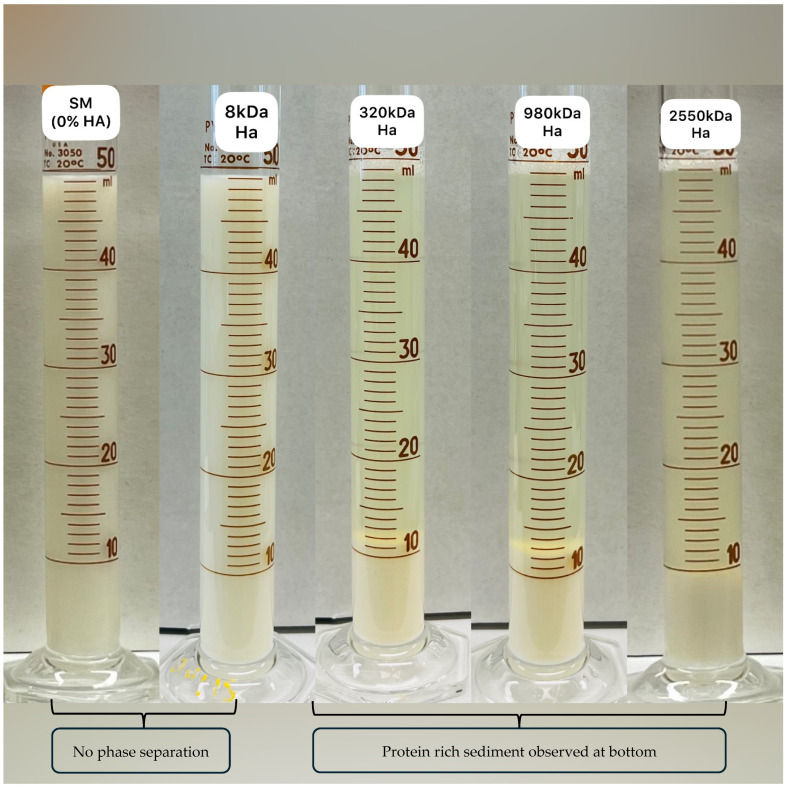
Visible protein phase separation as a function of skim milk sample treated with different molecular weight of HA at 0.25% *w*/*w* concentration.

The gravimetric protein phase separation method was used to study the impact on milk protein stability as a function of the increasing MW of HA. Figure 3 represents the visible phase separation of the protein-rich sediment layer observed after 48 h of storage. As per the image, there was no separate layer observed in the control and 8 kDa HA-treated samples. Increasing the MW from 8 kDa to 2550 kDa of HA led the samples to have visible phase separation, though the sediment layer in each of the three treatment samples was similar. Protein phase separation data are displayed in Table 3 which support the observed phase separation (Figure 3). HA with 8 kDa had no significant (*p* > 0.05) effect on protein stability as compared to the control, which indicates the inability of low MW polysaccharides to form an aggregate in the milk system. Moreover, there was no significant (*p* > 0.05) difference observed in the % protein phase separation for the 320 kDa, 980 kDa, and 2550 kDa HA treatment samples (61.54 ± 1.51, 66.74 ± 5.79, 66.04 ± 7.40 respectively). However, values increased significantly (*p* < 0.05) as compared to the control and 8 kDa treatment samples (5.425 ± 2.575, 4.50 ± 0.00). Sutariya and Salunke [9] reported 61.3 ± 0.4% protein phase separation in whole milk sample mixed with 0.25% 1550 kDa HA. The mechanism behind phase separation in high MW HA could be attributed to the depletion flocculation phenomenon which is driven by the entropic effects in the presence of non-adsorbing polymer. Non-adsorbing polymers increase the total entropy of the system, which causes an increase in the interaction between colloidal particles. As a result, colloidal particles tend to flocculate or aggregate [9,33]. 

### 3.4. Heat Coagulation Time Test (HCT)

**Figure 4:**
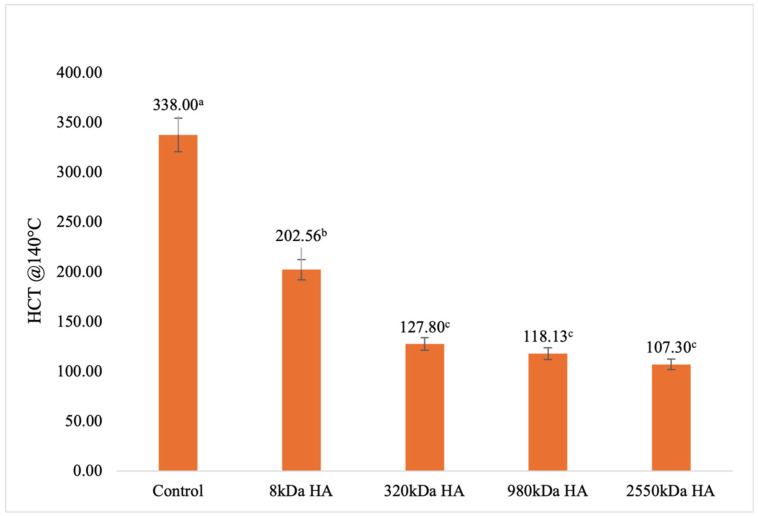
HCT @ 140 °C as a function skim milk samples treated with different molecular weights of HA 0.25% *w*/*w* concentration. Error bars are the standard error of mean for triplicate analysis for each treatment. ^a–c^ Completely different superscript letters between the HCT values on column bar shows significant differences (*p* < 0.05).

In the present study, the heat stability of milk–HA mixtures as a function of different MW of HA was determined when subjected to a high temperature (140 °C), and the results are depicted in Figure 4. The average HCT of the control skim milk sample was 338 s, which was comparable with the value (395 s) reported by Sutariya and Salunke [9]. The addition of HA (except for 8 kDa) into the skim milk significantly (*p* < 0.05) reduced the heat stability as compared to the control; however, the differences in the HCT remained non-significant (*p* > 0.05) among the 320, 980, and 2550 kDa samples. HCT in a whole milk sample added with 0.25% concentration of 1550 kDa HA was 140 ± 5 s [9]. A possible mechanism behind reduced heat stability could be attributed to the water-binding property of HA. Since HA polymers bind with water in the milk environment, the concentration of the heat-sensitive components in the continuous serum phase (i.e., soluble salts, lactose) increases, and upon heating at 140 °C, these compounds destabilize the colloidal structure of casein micelle and cause heat-induced protein aggregation [34,35,36,37]. 

### 3.5. Water Holding Capacity (WHC)

WHC is an important property through different stages of processing and storage, indicating the abilities of the product to hold moisture. Table 3 represents the changes in %WHC of the treatment samples as an effect of adding different MW of HA. The addition of 8 kDa HA did not introduce any statistically significant (*p* > 0.05) change in the %WHC of the sample. Compared to the control, an obvious increase in %WHC was noticed as MW increased from 8 kDa to 2550 kDa. The reason behind increasing WHC in the matrix could be attributed to the ability of anionic polysaccharides to form hydrogen bonds with water molecules [38]. However, contradictory results were observed in the case of 980 kDa HA sample, and the reason behind this still demands further investigation. 

### 3.6. Oil Emulsifying Activity (EA) and Emulsion Stability (ES)

The addition of HA influenced oil emulsifying activity and stability. EA and ES indicate the capacity of the polymer to stabilize oil in water emulsions either by lowering surface tension or by changing bulk viscosity and resisting the breakdown of emulsion under processing conditions, respectively [39]. The results of EA and ES are displayed in Table 3. Based on the results, both EA and ES increased significantly as molecular weight increased compared to the control except for the 8 kDa sample. The lowest emulsion activity and stability in the 8 kDa sample could be attributed to the low viscosity of the sample [13]. The increasing EA in high molecular weight HA polymer is due to their structuring ability in continuous phases and the formation of a high viscoelastic gel network in the system. As an effect, the movement of trapped oil droplets in the gel network is restricted. Moreover, an increased molecular weight of polymer increased steric repulsion and provided superior stability against processing conditions by preventing the aggregation and flocculation of droplets [40,41]. 

### 3.7. Foaming Capacity (FC) and Foaming Stability (FS)

The foaming capacity and stability of four different molecular weights of HA polymer, each at 0.25% concentration, are displayed in Table 4. The foaming behavior of skim milk varied as molecular weight changed. The highest foaming capacity was observed for the control, 8 kDa, and 320 kDa HA treatment samples (100.00 ± 0.0, 103.33 ± 13.33, 96.66 ± 3.33), whereas the lowest was found for the 2550 kDa sample (53.33 ± 3.33). Both control and 8 kDa showed poor foam retention. At 0.5 h, the foam values (%) of the control samples were reduced to nearly half of the initial foaming capacity observed at 0 h, and by the 6 h mark, it had completely dissipated to 0%. Likewise, in the 8 kda samples, foam retention reduced gradually and reached 0% at the end of storage. In the 320 kDa and 980 kDa treatment samples, the foam value (%) was reduced slightly at the 1 h mark (90.00 ± 0.0 and 63.33 ± 6.67) and remained constant until 6 h of storage. However, at the end of 24 h, values were found to have declined slightly and reached 66.66 ± 14.53 and 60.00 ± 0.00, respectively. However, foam values (%) for both 320 kDa and 980 kDa at each storage time were found to be statistically insignificant (*p* > 0.05), which indicated the improved foam retention ability of HA polymers of 320 kDa and 980 kDa MW at 0.25% concentration. Although a significant difference (*p* < 0.05) was found in foam values (%) of 2550 kDa HA polymer at 24 h storage (40.00 ± 0.0) from 0 h storage (53.33 ± 3.33), the values declined gradually until the 4 h mark and remained static up to 24 h of storage. A similar trend in foam values (%) was reported by Sutariya and Salunke [13], where the foam value (%) in a skim milk sample treated with 0.25% of 1550 kDa HA was 60 ± 0 at 0 h, which slowly dropped to 33 ± 0 after storage for 24 h. A solution is often more capable of producing foams if it has a lower surface tension and viscosity [42]. Nevertheless, when it comes to foam stability, high interfacial viscoelasticity is preferred. This phenomenon could be linked with the results obtained for the rheological behavior of samples in Section 3.2. As the MW increased (980 kDa and 2550 kDa HA), apparent viscosity in the milk environment increased, forming a stable interpolymer gel network that provided long stability to the foam against coalescence. 

### 3.8. Analyses of Individual Protein Fractions by Capillary Gel Electrophoresis (CGE)

**Figure 5:**
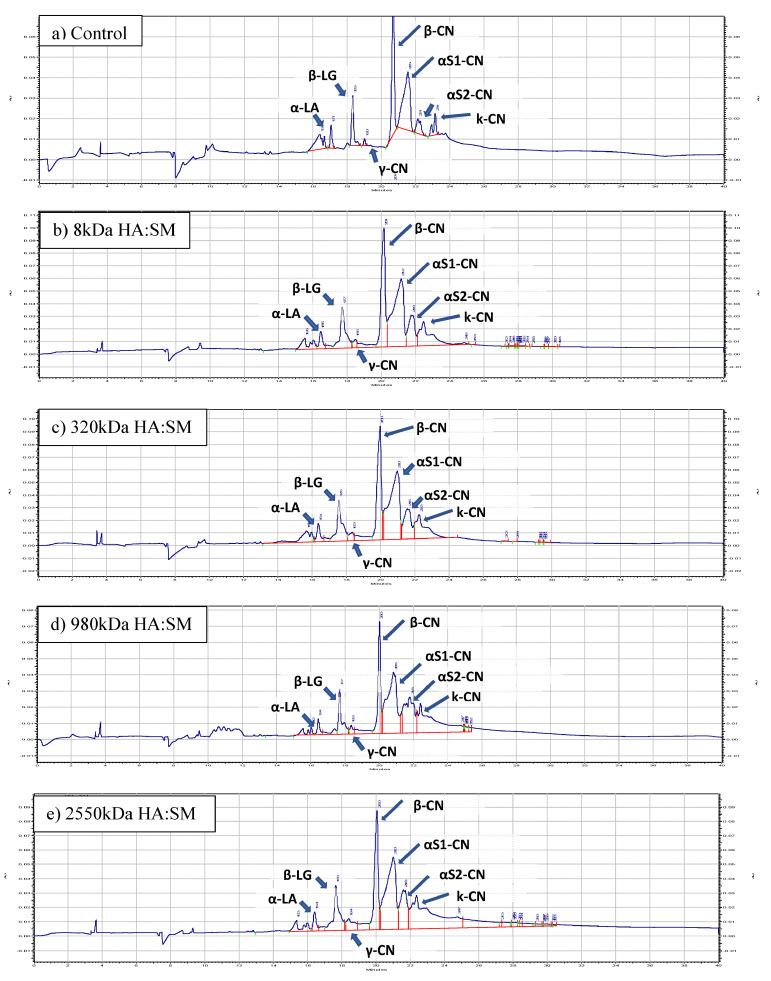
Typical electrophoreograms of skim milk treated with (**a**) Control, (**b**) 8 kDa HA, (**c**) 320 kDa HA, (**d**) 980 kDa HA, (**e**) 2550 kDa HA at 0.25% *w*/*w* concentration.

A CGE electropherogram of the control and treatment samples is shown in Figure 5, and peak analysis is collated in Table 5. In the CGE electropherogram (Figure 5) various CN fractions and whey protein fractions are identified based on the migration time. As per Figure 5, the CGE peak profile remained similar across all treatments, indicating that no significant modifications had occurred in protein fractions because of the HA. There was a broadening of the peak seen in κ-CN for the 980 kDa (Figure 5d) and 2550 kDa (Figure 5e) samples, indicating some interaction; however, data analysis (Table 5) revealed no significant differences even though the κ-CN values were lower. Table 5 represents % total peak areas quantified in various protein fractions for the control and treatment samples. In the control sample, the ratio for % peak area obtained for casein (CN) fractions (β-CN: αS1-CN: αS2-CN: κ-CN: γ-CN) was 3.9:4.4:0.8:0.7:0.13, whereas the ratio of β-LG: α-LA was 2.64. As per Salunke et al. [28], CN fractions (β-CN:αS1-CN:αS2-CN:κ-CN:γ-CN) and major serum proteins (SP) (β-LG:α-LA) obtained for skim milk were in the ratio of 4.1:4.0:1.0:0.7:0.3 and 2.42, respectively, whereas other researchers reported a CN fractions ratio of 4:4:1:1:0.4 and an SP ratio of 1.86 [43,44,45]. As per Table 5, there was no significant difference (*p* > 0.05) observed in all the protein fractions, including CN and WP fractions, of all the treatment samples. Similarly, % total CN and % total WP content were similar to that of the control.

### 3.9. Particle Size Analysis

Table 5 represents the mean particle size of protein present in the control (skim milk) and treatment samples in nm. The impact of HA on casein micelles is more apparent in skim milk as compared to whole milk containing fat because of the hindering effect of fat globules. The average particle size of the skim milk was 188.11 nm. Casein micelles have average hydrodynamic diameters between 150 and 200 nm while measured using dynamic light scattering [46]. It is evident from Table 5 that particle diameter in all treatment samples was non-significant (*p* > 0.05) as compared to the control, which represents the inability of HA molecules to bind with milk protein. The particle size results can be correlated with the results obtained in CGE analysis (Section 3.8), where no significant difference (*p* < 0.05) was observed in the total CN and total WP content of all samples. 

### 3.10. Quhiantification of Soluble and Insoluble Calcium Concentrations

The concentrations of soluble and insoluble calcium were determined to gain insights in any alterations in calcium ion concentrations resulting from the introduction of the anionic polysaccharide hyaluronic acid. The total calcium content in milk is approximately 30 mmol/L. Of this, ~10 mmol/L calcium exists in the milk serum phase, primarily in the form of calcium citrate, calcium phosphate, or free ions. The remaining ~20 mmol/L calcium is insoluble, residing within the colloidal phase as CCP in casein micelles or bound to phosphoserine residues [44]. Table 6 provides the quantified molar mass of calcium in both the soluble and insoluble phases. As per the results, the soluble calcium ion present in skim milk was 11.25 mmol/L, and the calculated concentration of insoluble calcium ion was 22.00 mmol/L. Notably, these findings align closely with the values reported by Fox et al. [44]. In addition, the results show that the addition of HA had no significant influence on calcium ion concentration as their *p* > 0.05, indicating no alteration in calcium ion equilibrium between the soluble and insoluble phases. These findings show the inability of HA to disintegrate casein micelles and transfer calcium ion from the colloidal (insoluble) phase to the serum (soluble) phase. 

### 3.11. Protein Microstructure Observation Using Confocal Laser Scanning Microscopy

**Figure 6:**
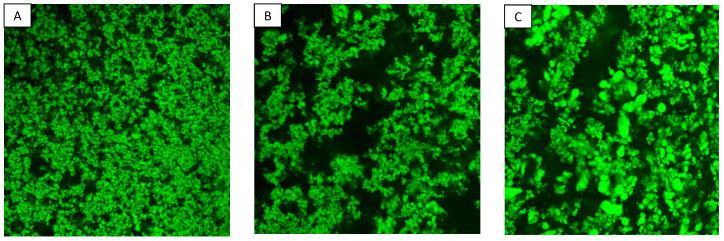
Confocal micrographs of skim milk treated with (**A**) 320 kDa HA, (**B**) 980 kDa HA, (**C**) 2550 kDa HA at 0.25% *w*/*w* concentration.

Figure 6 depicts the microstructural characteristics of skim milk incorporated with different molecular weights of HA. We were unable to capture micrographs of the control and 8 kDa samples due to the absence of gel formation. In Figure 6A–C, casein in skim milk with MWs of 320 kDa, 980 kDa, and 2550 kDa appears to form a three-dimensional protein network structure. Notably, low molecular weight HA (320 kDa) tends to create a continuous porous structure alongside small protein aggregate. In the micrographs obtained for 980 kDa HA, a slightly more open protein network structure characterized by the presence of visible large pores is seen. These larger pores (black pores) in the images are attributed to the water retention ability of added polysaccharide. As the MW of HA increases to 2550 kDa, a somewhat denser protein structure emerges, with irregular protein clumps with less visible pores as compared to 980 kDa HA. The possible reason behind irregular protein aggregates could be attributed to protein phase separation, although samples were vortexed during preparation. There were some changes seen in the κ-CN peak of the CGE electropherogram of the 980 kDa and 2550 kDa samples as reported in Section 3.8. The micrograph results revealed that 8 kDa HA did not induce gel formation in the milk at a 0.25% addition level. Moreover, as the MW of HA increased, protein structure tended to become denser in the presence of larger pores. These findings are inconsistent with the results obtained for water holding capacity, as discussed in Section 3.5. 

## 4. Conclusions

This research elucidates the impact of varying molecular weights of hyaluronic acid on the functional and rheological characteristics of skim milk. Based on the results, it can be concluded that addition of 8 kDa, 320 kDa, 980 kDa, and 2550 kDa HA significantly impacted the functional properties of skim milk when added at concentration of 0.25% *w*/*w*. With an increase in MW, an increase in the apparent viscosity and shear thinning behavior of skim milk was observed. Frequency sweep results represented higher loss modulus over storage modulus for all samples, which indicates the formation of a weak viscoelastic-solid gel network in the milk environment mainly through interparticle polymer interaction. Further, HA of all MW except 8 kDa tends to increase water holding capacity, emulsification properties, and foaming abilities. Since the 8 kDA HA had no significant influence on the milk’s functionality, it could be incorporated into milk, whey, and other beverages to impart health benefits without inducing excessive viscosity and maintaining product attributes such as color, flavor, texture, and turbidity. Also, no significant results were obtained for the viscosity, protein phase separation, and heat stability of SM with a 0.25% concentration of 980 kDa and 2550 kDa HA. This suggests that HA with a broad range of molecular weights, spanning from 980 kDa to 2550 kDa, could be used in its crude form without specific molecular weight-based fractionations during production. This would be beneficial in lowering the production costs associated with high molecular weight HA. From CGE and particle size, it could be stated that the\functionality of HA polymers in skim milk environment is largely via water binding as it did not show any structural changes in protein fractions and simultaneously, addition of HA did not change hydrodynamic diameter of particles. Microimaging of protein structures revealed the difference in protein structure as an effect of difference in MW and water retention ability. Although, a thorough understanding of interaction among milk proteins, HA and their gel formation dynamics could be explored using pure casein and whey protein isolate as treatments added with HA having different molecular weights. These findings hold the potential to advance the utilization of HA in dairy products that necessitates enhanced water retention and textural properties. High molecular weight HA significantly impacts the functional properties of skim milk and can be incorporated as a food additive in milk products such as yogurt, Ice cream, and processed cheese. Moreover, future research could be done on synergistic impact of different molecular weight HA at various concentration with/or without using other different types of hydrocolloids on milk and milk products functional properties.

## Figures and Tables

**Table 1:** Mean composition of pasteurized skim milk.

% Composition				
Fat	Total Protein	Lactose	Total Solids	pH@ 25 °C
0.06 ± 0.04	3.74 ± 0.03	4.98 ± 0.01	9.50 ± 0.03	6.65 ± 0.05

All values are the mean of triplicate analyses ± the standard error of mean.

**Table 2:** Power law-derived consistency coefficient (K) and flow behavior index (n) of skim milk samples treated with different molecular weights of hyaluronic acid at 0.25% *w*/*w* concentration.

Parameter	Treatment				
	Control	8 kDa	320 kDa	980 kDa	2550 kDa
log *K* (Pa s^n^)	0.00 ± 0.00 ^b^	0.00 ± 0.00 ^b^	0.02 ± 0.00 ^b^	0.92 ± 0.33 ^a^	0.71 ± 0.01 ^a^
n (-)	1.33 ± 0.04 ^a^	1.33 ± 0.03 ^a^	0.96 ± 0.02 ^b^	0.57 ± 0.04 ^c^	0.54 ± 0.02 ^c^

All values are the mean of triplicate analyses ± the standard error of mean. ^a–c^ Completely different superscript letters in row shows significant differences (*p* < 0.05).

**Table 3:** Water holding capacity (% WHC), Emulsion activity (% EA), Emulsion stability (%ES), % Protein phase separation (PPS) of skim milk samples treated with different molecular weights of hyaluronic acid at 0.25% *w*/*w* concentration.

Treatment					
Parameter	Control	8 kDa	320 kDa	980 kDa	2550 kDa
WHC	0.00 ± 0.00 ^c^	0.15 ± 0.03 ^c^	15.00 ± 0.37 ^b^	17.08 ± 1.00 ^a^	14.60 ± 0.22 ^b^
EA	40.00 ± 0.00 ^c^	39.58 ± 0.42 ^c^	55.83 ± 3.52 ^b^	89.17 ± 1.54 ^a^	95.83 ± 0.83 ^a^
ES	38.00 ± 1.23 ^c^	37.50 ± 1.71 ^c^	45.00 ± 3.16 ^c^	81.00 ± 4.40 ^b^	95.00 ± 0.00 ^a^
PPS	5.43 ± 2.58 ^b^	4.50 ± 0.00 ^b^	61.54 ± 1.51 ^a^	66.74 ± 5.79 ^a^	66.04 ± 7.40 ^a^

All values are the mean of triplicate analyses ± the standard error of mean. ^a–c^ Completely different superscript letters in a row show significant differences (*p* < 0.05).

**Table 4:** Foaming capacity and stability of milk samples treated with different molecular weights of hyaluronic acid at 0.25% *w*/*w* concentration.

Parameter	Time Points	Treatment				
		Control	8 kDa	320 kDa	980 kDa	2550 kDa
% Foam capacity	0 h	100.00 ± 0.00 ^abA^	103.33 ± 13.33 ^aA^	96.66 ± 3.33 ^ab^	63.33 ± 6.66 ^bc^	53.33 ± 3.33 ^cA^
% Foam stability (Foam retention)	0.5 h	45.00 ± 5.00 ^bAB^	76.66 ± 13.33 ^abA^	96.66 ± 3.33 ^a^	66.66 ± 0.00 ^ab^	50.00 ± 8.82 ^bAB^
	1 h	40.00 ± 10.00 ^bAB^	63.33 ± 8.82 ^abABC^	90.00 ± 5.77 ^a^	63.33 ± 6.67 ^ab^	46.66 ± 3.33 ^bAB^
	1.5 h	35.00 ± 15.00 ^bAB^	56.66 ± 3.33 ^bABC^	90.00 ± 5.77 ^a^	63.33 ± 6.67 ^ab^	46.66 ± 3.33 ^bAB^
	2 h	25.00 ± 15.00 ^cAB^	43.33 ± 6.66 ^bcABC^	90.00 ± 5.77 ^a^	63.33 ± 6.66 ^ab^	46.66 ± 3.33 ^bcAB^
	4 h	10.00 ± 10.00 ^cAB^	30.00 ± 11.55 ^bcBC^	90.00 ± 5.77 ^a^	63.33 ± 6.67 ^ab^	43.33 ± 3.33 ^bcAB^
	6 h	0.00 ± 0.00 ^cB^	23.33 ± 14.53 ^cBC^	90.00 ± 5.77 ^a^	63.33 ± 6.67 ^ab^	40.00 ± 0.00 ^bcB^
	18 h	0.00 ± 0.00 ^cB^	6.66 ± 6.67 ^cC^	83.33 ± 12.02 ^a^	60.00 ± 10.00 ^ab^	40.00 ± 0.00 ^bcB^
	24 h	0.00 ± 0.00 ^bcB^	0.00 ± 0.00 ^cC^	66.66 ± 14.53 ^a^	60.00 ± 10.00 ^a^	40.00 ± 0.00 ^abB^

All values are the mean of triplicate analyses ± the standard error of mean. ^a–c^ Completely different superscript letters in a row shows significant differences (*p* < 0.05). ^A–C^ Completely different superscript letters in a column shows significant differences (*p* < 0.05).

**Table 5:** Particle size (nm) of protein and % area of protein fractions observed in the capillary gel electrophoresis of skim milk samples treated with different molecular weights of hyaluronic acid at 0.25% *w*/*w* concentration.

Parameters		Control	8 kDa	320 kDa	980 kDa	2550 kDa
Particle size (nm)		188.11 ± 1.76	190.28 ± 1.77	199.28 ± 0.51	194.56 ± 3.49	190.70 ± 4.35
%Area	β-CN	33.36 ± 0.00	26.63 ± 1.85	27.31 ± 2.58	26.29 ± 1.51	28.52 ± 1.30
	αS1-CN	37.50 ± 0.00	36.50 ± 0.97	38.20 ± 2.06	39.29 ± 1.39	36.05 ± 1.48
	αS2-CN	6.57 ± 0.00	9.98 ± 0.52	8.87 ± 1.45	10.23 ± 1.42	7.96 ± 0.61
	k-CN	6.05 ± 0.00	8.29 ± 1.30	7.99 ± 1.64	5.03 ± 1.43	5.81 ± 1.35
	γ-CN	1.08 ± 0.00	1.66 ± 0.34	1.80 ± 0.52	2.50 ± 0.66	2.36 ± 0.50
	α-LA	4.24 ± 0.00	3.64 ± 0.28	3.12 ± 0.23	3.76 ± 0.39	4.13 ± 0.17
	β-LG	11.20 ± 0.00	13.24 ± 0.72	12.59 ± 1.71	12.85 ± 1.72	15.15 ± 0.28
	%Total CN	84.56 ± 0.00	83.06 ± 0.96	84.18 ± 1.97	83.34 ± 2.09	80.71 ± 0.40
	% Total WP	15.40 ± 0.00	16.88 ± 0.96	15.71 ± 1.92	16.61 ± 2.09	19.27 ± 0.42

All values are the mean of triplicate analyses ± the standard error of mean.

**Table 6:** Mass of soluble calcium (mmol/L) and insoluble calcium (mmol/L) in skim milk samples treated with different molecular weights of hyaluronic acid at 0.25% *w*/*w* concentration.

Treatments	Mass of Soluble Calcium in Serum (mmol/L)	Mass of Insoluble Calcium in Sediment (Calculated) (mmol/L)
Control	11.25 ± 0.48	22.00 ± 1.47
8 kDa	11.33 ± 0.84	22.50 ± 0.99
320 kDa	10.50 ± 0.76	23.33 ± 0.80
980 kDa	10.83 ± 0.48	23.33 ± 0.80
2550 kDa	10.66 ± 0.42	23.33 ± 1.26

All values are the mean of triplicate analyses ± the standard error of mean.

## Data Availability

The original contributions presented in the study are included in the article, further inquiries can be directed to the corresponding author.

## References

[1] Necas J., Bartosikova L., Brauner P., Kolar J. (2008). Hyaluronic acid (hyaluronan): A review. Vet. Med..

[2] Boeriu C.G., Springer J., Kooy F.K., van den Broek L.A., Eggink G. (2013). Production methods for hyaluronan. Int. J. Carbohydr. Chem..

[3] Cheng F., Gong Q., Yu H., Stephanopoulos G. (2016). High-titer biosynthesis of hyaluronic acid by recombinant Corynebacterium glutamicum. Biotechnol. J..

[4] Sheng J., Ling P., Wang F. (2015). Constructing a recombinant hyaluronic acid biosynthesis operon and producing food-grade hyaluronic acid in Lactococcus lactis. J. Ind. Microbiol. Biotechnol..

[5] Sze J., Brownlie J., Love C. (2016). Biotechnological production of hyalu-ronic acid: A mini review. 3 Biotech.

[6] Zając M., Kulawik P., Tkaczewska J., Migdał W., Filipczak-Fiutak M., Fiutak G. (2017). The effect of hyaluronic acid addition on the properties of smoked homogenised sausages. J. Sci. Food Agric..

[7] Mazzucco A. (2019). Hyaluronic acid: Evaluation of efficacy with different molecular weights. Int. J. Chem. Res.

[8] Food and Drug Administration (2020). GRAS Notice: GRN 976: Intended for Use as an Ingredient in Fruit Drinks/Ades, Carbonated Soft Drinks, Candy, Milk Drinks, Yogurt, and Ready-to-Eat Cereals at Levels Ranging from 40–60 mg/Serving. https://www.cfsanappsexternal.fda.gov/scripts/fdcc/?set=GRASNotices&id=976.

[9] Sutariya S.G., Salunke P. (2022). Effect of hyaluronic acid on milk properties: Rheology, protein stability, acid and rennet gelation properties. Food Hydrocoll..

[10] Oe M., Mitsugi K., Odanaka W., Yoshida H., Matsuoka R., Seino S., Kanemitsu T., Masuda Y. (2014). Dietary hyaluronic acid migrates into the skin of rats. Sci. World J..

[11] Tan C., Yao X., Jafari S.M., Sun B., Wang J. (2023). Hyaluronic acid-based nanodelivery systems for food bioactive compounds. Trends Food Sci. Technol..

[12] Wang N., Cheng J., Jiang Y., Meng Y., Zhang K., Ban Q., Wang X. (2023). Emulsions stabilised by casein and hyaluronic acid: Effects of high intensity ultrasound on the stability and vitamin E digestive characteristics. Ultrason. Sonochem..

[13] Sutariya S.G., Salunke P. (2023). Effect of Hyaluronic Acid and Kappa-Carrageenan on Milk Properties: Rheology, Protein Stability, Foaming, Water-Holding, and Emulsification Properties. Foods.

[14] Zhong W., Li C., Diao M., Yan M., Wang C., Zhang T. (2021). Characterization of interactions between whey protein isolate and hyaluronic acid in aqueous solution: Effects of pH and mixing ratio. Colloids Surf. B Biointerfaces.

[15] Chon J.-W., Kim B., Seo K.-H., Bae D., Jeong D., Song K.-Y. (2020). Physiochemical and organoleptic properties of kefir containing different concentrations of hyaluronic acid: A preliminary study. J. Dairy Sci. Biotechnol..

[16] Allison D.D., Grande-Allen K.J. (2006). Hyaluronan: A powerful tissue engineering tool. Tissue Eng..

[17] Fagien S., Cassuto D. (2012). Reconstituted injectable hyaluronic acid: Expanded applications in facial aesthetics and additional thoughts on the mechanism of action in cosmetic medicine. Plast. Reconstr. Surg..

[18] Kogan G., Šoltés L., Stern R., Gemeiner P. (2007). Hyaluronic acid: A natural biopolymer with a broad range of biomedical and industrial applications. Biotechnol. Lett..

[19] Jagannath S., Ramachandran K. (2010). Influence of competing metabolic processes on the molecular weight of hyaluronic acid synthesized by Streptococcus zooepidemicus. Biochem. Eng. J..

[20] Tammi R.H., Kultti A., Kosma V.-M., Pirinen R., Auvinen P., Tammi M.I. (2008). Hyaluronan in human tumors: Pathobiological and prognostic messages from cell-associated and stromal hyaluronan. Seminars in Cancer Biology.

[21] Hooi R., Barbano D., Bradley R., Budde D., Bulthaus M., Chettiar M., Lynch J., Reddy R. (2004). Chapter 15 chemical and physical methods. Standard Methods for the examination of dairy products.

[22] Sutariya S.G., Huppertz T., Patel H.A. (2017). Influence of milk pre-heating conditions on casein–whey protein interactions and skim milk concentrate viscosity. Int. Dairy J..

[23] Kasinos M., Karbakhsh R.R., Van der Meeren P. (2015). Sensitivity analysis of a small-volume objective heat stability evaluation test for recombined concentrated milk. Int. J. Dairy Technol..

[24] Sutariya S., Patel H. (2017). Effect of hydrogen peroxide on improving the heat stability of whey protein isolate solutions. Food Chem..

[25] Li R., Czaja T.P., Glover Z.J., Ipsen R., Jæger T.C., Rovers T.A., Simonsen A.C., Svensson B., van den Berg F., Hougaard A.B. (2022). Water mobility and microstructure of acidified milk model gels with added whey protein ingredients. Food Hydrocoll..

[26] Benvenutti L., Zielinski A.A.F., Ferreira S.R.S. (2022). Subcritical water extraction (SWE) modified by deep eutectic solvent (DES) for pectin recovery from a Brazilian berry by-product. J. Supercrit. Fluids.

[27] Cano-Medina A., Jiménez-Islas H., Dendooven L., Herrera R.P., González-Alatorre G., Escamilla-Silva E.M. (2011). Emulsifying and foaming capacity and emulsion and foam stability of sesame protein concentrates. Food Res. Int..

[28] Salunke P., Marella C., Metzger L.E. (2021). Microfiltration and ultrafiltration process to produce micellar casein and milk protein concentrates with 80% crude protein content: Partitioning of various protein fractions and constituents. Dairy.

[29] Lin L., Wong M., Deeth H., Oh H. (2018). Calcium-induced skim milk gels using different calcium salts. Food Chem..

[30] Patton J., Reeder W. (1959). Method for Titrating Calcium. U.S. Patent.

[31] Pearce K. (1977). The complexometric determination of calcium in dairy products. N. Z. J. Dairy Sci. Technol..

[32] Zhang Y., Xu X., Xu J., Zhang L. (2007). Dynamic viscoelastic behavior of triple helical Lentinan in water: Effects of concentration and molecular weight. Polymer.

[33] de Bont P.W., van Kempen G.M., Vreeker R. (2002). Phase separation in milk protein and amylopectin mixtures. Food Hydrocoll..

[34] Nayak S., Makrariya A., Singh R., Patel A., Sindhu J., Patil G., Tomar P. (2004). Heat stability and flow behaviour of buffalo milk added with corn starch. Food Hydrocoll..

[35] Tziboula A., Muir D.D. (1993). Milk protein-carbohydrate interactions. Int. Dairy J..

[36] Singh H., Creamer L. (1992). Heat stability of milk. Advanced Dairy Chemistry-1: Proteins.

[37] Tziboula A., Muir D.D. (1993). Effect of starches on the heat stability of milk. Int. J. Food Sci. Technol..

[38] Pang Z., Deeth H., Bansal N. (2015). Effect of polysaccharides with different ionic charge on the rheological, microstructural and textural properties of acid milk gels. Food Res. Int..

[39] Wilde P., Mackie A., Husband F., Gunning P., Morris V. (2004). Proteins and emulsifiers at liquid interfaces. Adv. Colloid Interface Sci..

[40] Tang Q., Huang G. (2022). Improving method, properties and application of polysaccharide as emulsifier. Food Chem..

[41] Costa C., Medronho B., Filipe A., Mira I., Lindman B., Edlund H., Norgren M. (2019). Emulsion formation and stabilization by biomolecules: The leading role of cellulose. Polymers.

[42] Bureiko A., Trybala A., Kovalchuk N., Starov V. (2015). Current applications of foams formed from mixed surfactant–polymer solutions. Adv. Colloid Interface Sci..

[43] Walstra P., Jenness R. (1984). Dairy Chemistry & Physics.

[44] Fox P.F., Mcsweeney P.L., Paul L. (1998). Dairy Chemistry and Biochemistry.

[45] Farrell Jr H., Jimenez-Flores R., Bleck G., Brown E., Butler J., Creamer L., Hicks C., Hollar C., Ng-Kwai-Hang K., Swaisgood H. (2004). Nomenclature of the proteins of cows’ milk—Sixth revision. J. Dairy Sci..

[46] Sinaga H., Bansal N., Bhandari B. (2017). Effects of milk pH alteration on casein micelle size and gelation properties of milk. Int. J. Food Prop..

